# Effects of physical activity on aggressive behavior of college students: The chain mediating role of self-esteem and self-control

**DOI:** 10.1097/MD.0000000000046779

**Published:** 2025-12-26

**Authors:** Siqiang Guo, Huaying Fu, Kelei Guo

**Affiliations:** a School of Physical Education and Health, Zhaoqing University, Zhaoqing, PR China; b College of Economics and Management, Zhaoqing University, Zhaoqing, PR China.

**Keywords:** aggressive behavior, mediation analysis, mental health, physical activity, school violence, self-control, self-esteem

## Abstract

Aggressive behavior has emerged as a critical concern in the global educational context, posing risks for interpersonal conflicts, psychological distress, and potential violence. This study aimed to investigate the relationship between physical activity and aggressive behavior among college students, while also examining the mediating roles of self-esteem and self-control.

## 1. Introduction

Aggressive behavior encompasses intentional actions or tendencies that harm the physical and psychological well-being of others.^[1]^ It serves as an external manifestation of the underlying psychological issues and neurological disorders.^[2]^ In 2019, UNESCO published a research report titled “Behind the numbers: ending school violence and bullying,” highlighting the global concern regarding frequent incidents of aggressive behaviors on campuses, which profoundly impact the mental health of college students.^[3]^ Studies have indicated that approximately 83.93% of college students exhibit moderate-to-high levels of aggression.^[4]^ The primary aim of this study is to elucidate a comprehensive psychological mechanism in which sports activities serve as the initial antecedent, self-esteem functions as a guiding variable, self-control operates as the central mediator, and aggressive behavior emerges as the outcome. By employing rigorous empirical data, this research provides a scientifically grounded basis for universities to develop targeted behavioral intervention strategies. Notably, this study pioneers the application of a chain mediation model to delineate the sequential influence pathway – namely, from sports activities to cognitive-behavioral factors and ultimately to aggressive behavior – thus addressing the oversimplified mediating mechanisms observed in prior research. This advancement offers a novel theoretical framework for understanding the positive impact of physical activity on mental health.

The findings provide theoretical guidance for mitigating or ameliorating aggressive behaviors among college students through physical activity physical activity encompasses a spectrum of bodily movements that lead to energy expenditure through the contraction of skeletal muscles.^[5]^ Extensive evidence has established the significant role of physical activity in aggression prevention.^[6,7]^ College students with higher levels of physical activity exhibited lower levels of aggressive behavior than those with lower levels of physical activity.^[8]^ Engaging in physical activity not only enhances personal social skills, releases excess energy, improves mental well-being, and promotes emotional regulation but also serves as a means to mitigate a portion of aggressive behaviors.^[9]^ We propose hypothesis – hypothesis 1: physical activity negatively predicts aggressive behavior (H1).

Self-esteem refers to an individual’s evaluative perception of oneself, which is shaped by interactions with others and the surrounding environment and serves as a psychological mechanism for adaptation and maintaining mind-body harmony.^[10]^ A strong positive correlation exists between physical activity and self-esteem, with physical activity exerting a beneficial moderating influence on self-esteem.^[11]^ Participation in physical activity not only fosters improved interpersonal relationships and heightened self-efficacy, but also significantly impacts individuals’ holistic perception and evaluation of themselves.^[12]^

The association between self-esteem and aggressive behavior has garnered significant attention and sparked extensive debate, prompting researchers to explore 2 distinct perspectives. First, a negative correlation was observed among college students regarding the relationship between self-esteem and aggressive behavior, suggesting that an individual’s level of aggression is somewhat influenced by their level of self-esteem.^[13]^ Second, increased aggression as associated with high self-esteem.^[14]^ This association may be attributed to the presence of risk-seeking traits and preference for challenging situations commonly observed among individuals with elevated self-esteem. Therefore, we propose our second hypothesis – hypothesis 2: self-esteem mediates the relationship between physical activity and aggressive behavior.

Self-control is the process by which people adjust their behavior to achieve a desired, long-term goal state when experiencing response conflict, which encompasses both inhibitory and proactive strategies of self-control.^[15]^

Physical activity has been found to foster cultivation of self-control among college students.^[16]^ Elevated levels of self-control are indicative of reduced aggressive behavior, including anger expression, aggressive tendencies, rumination about experiences of anger, and antisocial conduct.^[17]^ Studies in the field of emotional recovery and regulation suggest that engagement in physical activity does not lead to a decrease or increase in negative emotions. Instead, it facilitates more effective recuperation and regulation when confronted with these emotions.^[18]^

Recent research has revealed that college students with low self-control demonstrate an increased propensity for aggressive behavior.^[19]^ With good self-control, individuals can effectively cope with various events in daily life and promote social interaction and efficient functioning. Conversely, individuals with compromised self-control tend to exhibit undesirable behaviors such as violence and involvement in criminal activities.^[20]^ Individuals lacking self-control are more susceptible to engaging in physical or verbal aggression, experiencing anger and hostility, and exhibiting a negative correlation with aggressive behavior.^[21]^ Therefore, we propose our third hypothesis – hypothesis 3: self-control mediates the relationship between physical activity and aggressive behavior.

Previous studies have found a positive correlation between self-esteem and self-control. Individuals with higher self-esteem demonstrated greater accuracy in predicting and exhibiting self-control.^[22]^ Self-esteem reflects an individual’s overall appraisal or attitude, whereas self-control represents the depth of their interaction with the environment. In threatening situations, individuals with low self-esteem experience a decrease in their regulatory abilities and subsequently exhibit reduced self-control. Conversely, individuals with high self-esteem can mitigate the impact of threatening situations on their levels of self-control.^[23]^ Therefore, we propose research hypothesis 4: self-esteem and self-control play chain-mediating roles between physical activity and aggressive behavior.

In summary, this study aims to achieve the following 4 objectives: verify the positive predictive effect of physical activity as a preventive measure against the aggressive behavior of college students; explore the mediating role of psychological self-esteem between physical activity and aggressive behavior of college students; investigate the mediating role of self-control between physical activity and aggressive behavior of college students; and prove that self-esteem and self-control play a chain-mediating role.

## 2. Materials and methods

### 2.1. Procedure and participants

Stratified cluster sampling was used to select 1000 students from 3 universities in Guangdong Province to complete the questionnaire survey. Invalid questionnaires with regular answers were deleted, and 931 valid questionnaires were obtained, with an effective recovery rate of 93.1%. The age range of the subjects was 18 to 22 years, with an average age of 19.77 ± 1.26, including 479 boys (51.5%) and 452 girls (48.5%). The participants were 274 freshmen, 254 sophomores, 235 juniors, and 168 seniors.

This study was approved by the Research Ethics Committee of the Zhaoqing University (No. 2023-0607-05). A collective test was then conducted. Before the survey, students were informed that the questionnaire was anonymous, emphasizing voluntary filling. The content is strictly confidential, and the results are only for scientific research. All questionnaires were collected on a spot. All participants were informed of the purpose and characteristics of the study and provided written informed consent. The participants were professionally trained college students.

### 2.2. Measures and instruments

#### 2.2.1. Physical activity

We used the life orientation test developed by Chen and revised by Wu ^[24]^ to measure physical activity. The scale consisted of 8 items divided into 2 dimensions. Items 1 to 4 measure physical exercise commitment, which are all positively scored; items 5 to 8 measure physical exercise persistence, where items 5, 7, and 8 are positively scored and 6 is negatively scored. Responses were evaluated using a 5-point Likert scale, with 1 indicating “strongly disagree” and 5 indicating “strongly agree.” The total score reflects the participants’ level of physical activity, with higher levels of physical activity and lower scores indicating higher and lower levels of physical activity, respectively. In this study, Cronbach’s α coefficient of the scale was 0.92.

#### 2.2.2. Self-esteem

We used the self-esteem scale developed by Rosenberg.^[25]^ A total of 10 items were included, employing a 4-point Likert scale ranging from 1 (strongly disagree) to 4 (strongly agree). Among these items, 3, 5, 8, 9, and 10 were reverse scored. The scores ranged from 10 to 40, with higher scores indicating elevated levels of self-esteem. Considering the cultural disparities between China and Western countries, question number 8 was positive scored in this particular study. The Cronbach’s α coefficient for this scale yielded a high value of 0.88.

#### 2.2.3. Self-control

In this study, the Chinese version of the self-control scale compiled by Tangney and revised by Tan and Guo.^[26]^ The scale comprises 19 questions, covering 5 dimensions, including impulse control (e.g., “I’m a bit of a spendthrift.”), healthy habits (e.g., “I am lazy.”), resisting temptation (e.g., “I can resist temptation fairly well.”) focusing on work (e.g. “I can work efficiently for a long-term goal.”) and restraint of entertainment (e.g., “I surf the Internet too much.”) It adopts the Likert 5-point scoring method, with a scoring range from 1 (strongly disagree) to 5 (strongly agree), and the higher the score, the stronger the self-control ability. In this study, Cronbach’s α coefficient of the scale was 0.91.

#### 2.2.4. Aggressive behavior

The present study employed the Buss-Perry Aggression Questionnaire, developed by Bryant and Smith and revised by Zhang et al,^[27]^ which consists of 4 dimensions: physical aggression, verbal aggression, anger, and hostility, encompassing a total of 12 items. Participants’ responses were evaluated on a 5-point Likert scale ranging from “strongly disagree” to “strongly agree,” with higher scores indicating heightened levels of aggression. The Cronbach’s α coefficient for this scale yielded a high value of 0.85.

### 2.3. Statistical analysis

Pearson correlation analysis and mediation effect tests were conducted using SPSS (version 26.0; IBM Corp, Armonk) and PROCESS macro.

## 3. Results

### 3.1. Common method deviation test and normal distribution test

The common method bias was examined using the Harman 1-way test, which showed that 8 factors had eigenvalues >1. The first factor explained only 30.41% of the variance, which is less than the critical criterion of 40%. Therefore, we concluded that there was no serious problem with the common method bias.

### 3.2. The demographic characteristics

Table 1 indicates that males exhibit significantly higher levels of physical activity and self-control compared to females. Conversely, although females demonstrate higher levels of aggressiveness and self-esteem, these differences are not statistically significant.

**Table 1:** Differences in gender.

Variable	Gender	Number (%)	*M*	SD	*t*	*P*
Physical activity	Male	452 (51.5%)	30.745	6.148	8.170	.010
	Female	452(48.5%)	27.341	6.566		
Aggressive behavior	Male	479(51.5%)	25.697	7.375	−.946	.043
	Female	452 (48.5%)	26.137	6.785		
Self-esteem	Male	479(51.5%)	31.048	4.865	−.038	.156
	Female	452 (48.5%)	31.059	4.471		
Self-control	Male	479(51.5%)	64.693	13.575	2.348	.002
	Female	452 (48.5%)	62.708	12.126		

N = 931.

SD = standard deviation.

### 3.3. Descriptive statistics and correlation analysis

According to Table 2, the analysis showed that physical activity was significantly positively correlated with self-esteem and self-control and significantly positively correlated with negative aggressive behavior. Self-esteem was significantly positively correlated with self-control and significantly positively correlated with negative aggressive behavior, self-control was also significantly negatively correlated with aggressive behavior.

**Table 2:** Means, standard deviations, and correlations among variables.

Variable	*M*	SD	Physical activity	Aggressive behavior	Self-esteem	Self-control
Physical activity	29.092	6.575	1			
Aggressive behavior	25.911	7.094	−0.422*	1		
Self-esteem	31.054	4.676	0.467*	−0.526*	1	
Self-control	63.729	12.923	0.513*	−0.630*	0.605*	1

N = 931.

SD = standard deviation.

* *P* < .01

### 3.4. Mediation effect analysis and mediation effect test

Based on the correlation analysis, we subsequently tested the chain mediation model, with results presented in Table 3. Initially, demographic variables (i.e., gender and age) were controlled to examine the direct relationship between physical activity and aggressive behavior. Before introducing the mediating variables, a significant direct pathway from physical activity to aggressive behavior was observed (β = −0.10, *t* = −3.2365, *P* < .001), supporting hypothesis 1.

**Table 3:** Analysis of regression relationship among variables.

Effect	Item	Effect	SE	*t*	*P*	LLCI	ULCI
Direct effect	Physical activity ⇒ aggressive behavior	−0.10	0.031	−3.236	.000	−0.160	−0.039
Indirect effect process	Physical activity ⇒ self-esteem	0.50	0.030	16.859	.000	0.443	0.560
	Physical activity ⇒ self-control	0.30	0.029	10.076	.000	0.237	0.352
	Self-esteem ⇒ self-control	0.47	0.028	16.520	.000	0.411	0.522
	Self-esteem ⇒ aggressive behavior	−0.20	0.032	−6.273	.000	−0.264	−0.138
	Self-control ⇒ aggressive behavior	−0.46	0.033	−14.006	.000	−0.524	−0.395
Total effect	Physical activity ⇒ aggressive behavior	−0.44	0.031	−14.442	.000	−0.504	−0.383

LLCI is the lower 95% limit for Bootstrap sampling and ULCI is the upper 95% limit for Bootstrap sampling.

N = 931.

CI = confidence interval, LL = lower limit, SE = standard error, UL = upper limit.

Next, a chain mediation analysis was conducted to explore the mediating roles of self-esteem and self-control in the relationship between physical activity and aggressive behavior. The study founded 3 significant indirect pathways. First, physical activity positively predicted self-esteem (β = 0.50, *t* = 16.859, *P* < .001), and self-esteem, in turn, negatively predicted aggressive behavior (β = −0.20, *t* = −6.273, *P* < .001), supporting hypothesis 2. Second, physical activity positively predicted self-control (β = 0.30, *t* = 10.076, *P* < .001), which subsequently negatively predicted aggressive behavior (β = −0.46, *t* = −14.006, *P* < .001), thereby supporting hypothesis 3. Finally, self-esteem was found to have a positive effect on self-control (β = 0.47, *t* = 16.520, *P* < .001), validating hypothesis 4.

**Figure 1.**
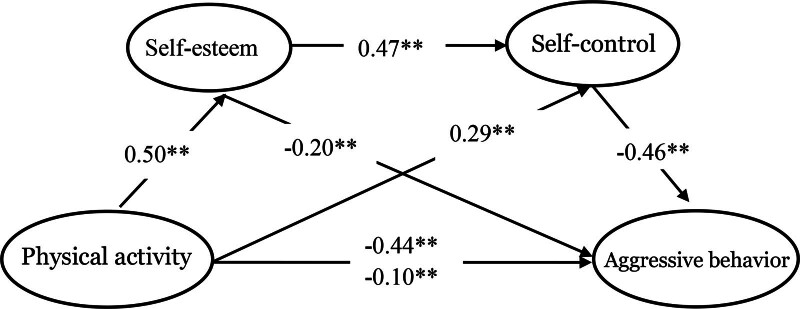
Chain mediation model – self-esteem and self-control play chain mediation model between physical activity and aggressive behavior. ** *P* < .01.

The deviation-corrected percentile Bootstrap method was employed with 5000 replications to test the mediation effects. According to Table 4, the 95% confidence intervals for the mediation pathways were as follows: for the pathway from physical activity to self-esteem and then too-aggressive behavior, the confidence interval was [−0.139, −0.067] with a mediation effect size of −0.101, accounting for 22.75% of the total effect; for the pathway from physical activity to self-control and then to aggressive behavior, the confidence interval was [−0.175, −0.101] with a mediation effect size of −0.1354, accounting for 30.54% of the total effect; and for the pathway from physical activity to self-esteem and then to self-control before affecting aggressive behavior, the confidence interval was [−0.137, −0.083] with a mediation effect size of −0.108, accounting for 24.24% of the total effect. As the 95% Bootstrap confidence intervals do not include zero, the mediating effects are statistically significant, with the mediating effect of self-esteem being the most pronounced. Finally, the chain mediation model of self-esteem and self-control between physical activity and aggressive behavior was obtained, as illustrated in Figure 1.

**Table 4:** Mediating effect test.

	Effect	Boot SE	95% CI		Ration of indirect to total effect
			LL	UL	
Total effect	−0.443	0.031	−0.504	−0.383	
Direct effect	−0.100	0.031	−0.160	−0.039	22.47%
Indirect effect					
Total indirect effect	−0.344	0.026	−0.396	−0.295	77.53%
Physical activity → self-esteem → aggressive behavior	−0.101	0.019	−0.139	−0.067	22.75%
Physical activity → self-control → aggressive behavior	−0.135	0.019	−0.175	−0.101	30.54%
Physical activity → self-esteem → self-control → aggressive behavior	−0.108	0.014	−0.137	−0.083	24.24%

The upper 95% limit for Bootstrap sampling.

CI = confidence interval, LL = lower limit, SE = standard error, UL = upper limit.

## 4. Discussion

### 4.1. Physical activity and aggressive behavior

In the present study, a significant negative correlation was observed between physical activity and aggression, consistent with the findings of a previous study^[7]^ thereby confirming hypothesis 1. It is widely acknowledged that college students, who are in a phase of mental imbalance and immaturity, exhibit heightened neurological excitability, but struggle with impulse control. Additionally, they possess emotional depth, but also demonstrate high emotional volatility. According to frustration-aggression theory, participation in physical activities contributes to enhancing individuals’ cognitive abilities and resilience towards frustration, facilitating stable emotional development while reducing the occurrence of aggressive behavior.^[28]^ Moreover, physical activity promotes the acquisition of social skills, dissipates excessive energy, improves emotion regulation capabilities, and effectively prevents and manages individual aggression by releasing pentup emotions.^[9]^

### 4.2. Independent mediating effects of self-esteem

The present study revealed that self-esteem serves as a mediating factor in the relationship between physical activity and aggressive behavior among college students, thereby validating hypothesis 2. This finding aligns with previous research indicating that engagement in physical activity positively influences self-esteem,^[11]^ while elevated self-esteem facilitates aggression prevention.^[13]^

On the 1 hand, Engagement in physical activity is positively associated with an individual’s level of self-esteem. According to Fredrickson’s broaden-and-build theory of positive emotions,^[29]^ the experience of positive emotions not only facilitates the expansion of an individual’s attention, cognitive, and behavioral capacities, but also contributes to the construction and enhancement of enduring personal resources.

On the other hand, participation in physical activity is itself an activity with positive self-changing qualities, wherein individuals continuously strive to enhance their athletic prowess and physical fitness while concurrently cultivating positive character traits, recognizing their inherent value, and garnering increased recognition and favorable evaluations from peers, educators, and parents. Conversely, college students who engage in less physical activity experience reduced adherence to social norms and a diminished sense of self-worth due to limited interpersonal communication and minimal exposure to the external world, consequently impacting their self-esteem.

### 4.3. Independent mediation effects of self-control

This study provides empirical evidence supporting Hypothesis 3 by demonstrating that self-control acts as a mediating factor in the relationship between physical activity and aggressive behavior. This finding is consistent with previous research,^[7]^ which consistently indicates a positive association between physical activity and self-control, as well as a significant negative correlation between self-control and aggressive behavior.

The research findings confirmed that engaging in physical activity has a significant positive impact on individuals’ self-control. According to theories related to self-control, when faced with complex physical activity scenarios, individuals must quickly adapt and adjust their cognitive strategies to accommodate the actions of peers and opponents, as well as changing circumstances. Furthermore, they need to develop more advanced cognitive functions through long-term practice to enhance their executive control.^[30]^ As physical activity intensity increases, an individual’s capacity for self-control is gradually exhausted, resulting in a state of physiological limitation in which self-control declines. Nevertheless, by persevering and exceeding this threshold, individuals can augment their level of self-regulation.

Self-control capacity is a significant and inverse predictor of aggressive behavior. According to energy modeling theory, engaging in long-term physical activity actively contributes to cultivating positive and optimistic coping styles, as well as fostering adaptive flexibility in response to unexpected stressful events. Ultimately, this cultivates enhanced self-regulation and reduces the occurrence of aggressive behavior.^[31]^ In the domain of behavioral decision making related to self-control, college students who exhibit elevated levels of self-discipline demonstrate an augmented capacity to resist external temptations (such as video games, monetary rewards, and food) by actively refraining from staying up late and consistently opting for behaviors or objectives that align with their long-term personal development goals. Conversely, college students with lower levels of self-control display varying degrees of anxiety when confronted with diverse temptations and academic pressures, leading them to employ maladaptive coping strategies (such as avoidance and aggression) to alleviate internal distress and pressure.^[32]^

### 4.4. Self-esteem and self-control chain mediation effects

This study constructed a chain-mediated model to examine the processes and mechanisms that link physical activity to aggressive behavior. Self-esteem and self-control play a mediating role in connecting physical activity with aggression. Regular engagement in physical activity enhances self-esteem, while high self-esteem significantly predicts self-control. Furthermore, independent of other factors, self-control also serves as a predictor of aggressive behavior. These findings support Hypothesis 4, which is consistent with previous research outcomes.^[33]^

The findings suggest a strong correlation between self-esteem and self-control. According to the energy model theory, prolonged engagement in physical activity promotes the development of positive and optimistic coping strategies as well as the ability to effectively handle various unexpected stressors, ultimately reducing aggressive behavior by enhancing self-control.^[31]^ Regular physical activity has been shown to positively impact self-esteem by promoting a favorable projection of personal image and enhancing feelings of competence. Moreover, it is worth noting that self-esteem serves as an effective coping mechanism against negative emotions by acting as a protective shield against various threats or stressors, while also preventing the depletion of psychological resources. Consequently, it fosters resilience in dealing with setbacks or failures, while simultaneously reducing vulnerability to negative emotional experiences.^[34]^

Therefore, the level of physical activity among college students is significantly negatively correlated with aggressive behavior, thereby contributing to the enhancement of individuals’ emotional well-being and life satisfaction, which in turn improves their self-esteem. Furthermore, individuals with high self-esteem are more inclined to engage in effective communication with their peers and exhibit a greater sense of optimism for the future, which can effectively prevent aggression by fostering self-control and promoting psychological well-being.

### 4.5. Limitations and prospects

In this study, we were unable to infer causal relationships between the variables. Future studies should adopt longitudinal follow-up and experimental intervention designs. In addition, in this study, we only considered the mediating effects of self-esteem and self-control. In fact, there may be other mediating variables, such as self-efficacy and peer relationships, that need to be further explored.

## 5. Conclusion

This study contributes to a better understanding of the effects of physical activity on aggressive behavior among college students. Physical activity, self-esteem, and self-control had a direct effect on aggressive behavior. Physical activity can not only directly influence aggression, but also indirectly influence aggression through the separate mediating roles of self-esteem and self-control, as well as the chain mediating roles of the 2, which further explains the reasons for the role of physical activity in college students’ aggression and is important for intervening in college students’ aggression.

## Acknowledgments

We would like to thank all the participants for their involvement in this study.

## Author contributions

**Conceptualization:** Kelei Guo.

**Data curation:** Kelei Guo.

**Formal analysis:** Huaying Fu.

**Project administration:** Kelei Guo.

**Writing – original draft:** Siqiang Guo, Huaying Fu.

**Writing – review & editing:** Siqiang Guo, Huaying Fu, Kelei Guo.
